# Increase in Acute Respiratory Illnesses Among Children and Adolescents Associated with Rhinoviruses and Enteroviruses, Including Enterovirus D68 — United States, July–September 2022

**DOI:** 10.15585/mmwr.mm7140e1

**Published:** 2022-10-07

**Authors:** Kevin C. Ma, Amber Winn, Heidi L. Moline, Heather M. Scobie, Claire M. Midgley, Hannah L. Kirking, Jennifer Adjemian, Kathleen P. Hartnett, Dylan Johns, Jefferson M. Jones, Adriana Lopez, Xiaoyan Lu, Ariana Perez, Cria G. Perrine, Andzelika E. Rzucidlo, Meredith L. McMorrow, Benjamin J. Silk, Zachary Stein, Everardo Vega, New Vaccine Surveillance Network Collaborators, Aron J. Hall, New Vaccine Surveillance Network Collaborators, Leila C. Sahni, Vasanthi Avadhanula, Natasha B. Halasa, Laura S. Stewart, Eileen J. Klein, Janet A. Englund, Geoffrey A. Weinberg, New York, Peter G. Szilagyi, Rangaraj Selvarangan, Jennifer E. Schuster, John V. Williams, Marian G. Michaels, Mary A. Staat, Christina Quigley

**Affiliations:** ^1^National Center for Immunization and Respiratory Diseases, CDC; ^2^Epidemic Intelligence Service, CDC; ^3^Center for Surveillance, Epidemiology, and Laboratory Services, CDC; ^4^ICF, Atlanta, Georgia; ^5^General Dynamics Information Technology, Inc., Falls Church, Virginia, ^6^InductiveHealth Informatics, Atlanta, Georgia.; Baylor College of Medicine and Texas Children’s Hospital, Houston, Texas; Baylor College of Medicine, Houston, Texas; Vanderbilt University Medical Center, Nashville, Tennessee; Vanderbilt University Medical Center, Nashville, Tennessee; Seattle Children’s Hospital, Seattle, Washington; Seattle Children’s Hospital, Seattle, Washington; University of Rochester School of Medicine and Dentistry, Rochester; University of Rochester School of Medicine and Dentistry, Rochester, New York and University of California at Los Angeles, Los Angeles, California; Children’s Mercy Kansas City, Kansas City, Missouri; Children’s Mercy Kansas City, Kansas City, Missouri; UPMC Children’s Hospital of Pittsburgh, Pittsburgh, Pennsylvania; UPMC Children’s Hospital of Pittsburgh, Pittsburgh, Pennsylvania; Cincinnati Children’s Hospital Medical Center, Cincinnati, Ohio; Cincinnati Children’s Hospital Medical Center, Cincinnati, Ohio

Increases in severe respiratory illness and acute flaccid myelitis (AFM) among children and adolescents resulting from enterovirus D68 (EV-D68) infections occurred biennially in the United States during 2014, 2016, and 2018, primarily in late summer and fall. Although EV-D68 annual trends are not fully understood, EV-D68 levels were lower than expected in 2020, potentially because of implementation of COVID-19 mitigation measures (e.g., wearing face masks, enhanced hand hygiene, and physical distancing) (*1*). In August 2022, clinicians in several geographic areas notified CDC of an increase in hospitalizations of pediatric patients with severe respiratory illness and positive rhinovirus/enterovirus (RV/EV) test results.* Surveillance data were analyzed from multiple national data sources to characterize reported trends in acute respiratory illness (ARI), asthma/reactive airway disease (RAD) exacerbations, and the percentage of positive RV/EV and EV-D68 test results during 2022 compared with previous years. These data demonstrated an increase in emergency department (ED) visits by children and adolescents with ARI and asthma/RAD in late summer 2022. The percentage of positive RV/EV test results in national laboratory-based surveillance and the percentage of positive EV-D68 test results in pediatric sentinel surveillance also increased during this time. Previous increases in EV-D68 respiratory illness have led to substantial resource demands in some hospitals and have also coincided with increases in cases of AFM (*2*), a rare but serious neurologic disease affecting the spinal cord. Therefore, clinicians should consider AFM in patients with acute flaccid limb weakness, especially after respiratory illness or fever, and ensure prompt hospitalization and referral to specialty care for such cases. Clinicians should also test for poliovirus infection in patients suspected of having AFM because of the clinical similarity to acute flaccid paralysis caused by poliovirus. Ongoing surveillance for EV-D68 is critical to ensuring preparedness for possible future increases in ARI and AFM.

ARI caused by EV-D68 primarily affects young children with varying severity. Typical signs and symptoms include cough, nasal congestion, wheezing, and dyspnea; infection can exacerbate asthma or RAD (*1*,*3*,*4*). Children with a history of asthma/RAD might be more likely to require medical care, although any child with ARI caused by EV-D68 can have severe illness (*3*,*4*). Importantly, EV-D68 is associated with AFM, a severe condition that can lead to muscle weakness and paralysis (*2*). Standard multiplex respiratory panels cannot distinguish between RVs and EVs or identify specific virus types. Thus, EV-D68 cases are likely undercounted because type identification is not routinely performed and reporting is not mandatory.^†^

Weekly data from three sources were analyzed for this report. First, weekly ED visits from week 1 of 2018 through week 37 of 2022 by children and adolescents aged <18 years from the National Syndromic Surveillance Program (NSSP) were assessed^§^; visits with ARI^¶^ and asthma/RAD** were identified, and quality control filters were applied to allow comparison across years.^††^ Second, weekly percentages of positive RV/EV test results from week 1 of 2014 through week 35 of 2022 were analyzed from the National Respiratory and Enteric Virus Surveillance System (NREVSS),^§§^ a network of 473 laboratories that passively report aggregated testing data. Third, RV/EV and EV-D68 detections were assessed among children and adolescents aged <18 years who visited an ED or were hospitalized for ARI within the New Vaccine Surveillance Network (NVSN)^¶¶^ during 2017–2022; the weekly percentages of pediatric patients with a positive RV/EV test result who also had a positive EV-D68 test result were characterized. For all platforms, descriptive analyses of longitudinal trends compared with previous years were conducted and stratified by age group and geographic region, where available. This activity was reviewed by CDC and was conducted consistent with applicable federal law and CDC policy.***

The percentage of ED visits among children and adolescents aged 0–4 and 5–17 years that were associated with ARI has been qualitatively elevated from week 15 through week 37 of 2022 (the endpoint of available data) compared with 2018–2020; levels were comparable with summer 2021, when respiratory syncytial virus circulation was elevated (Figure 1) (Supplementary Figure 1, https://stacks.cdc.gov/view/cdc/121524).^†††^ A more recent increase in the percentage of ED visits with ARI began on week 31 among both age groups. The percentage of ED visits associated with asthma/RAD in 2022 among children aged 0–4 years was qualitatively higher in all weeks from week 29 to 37 compared with the corresponding weeks during 2018–2021, and by week 37 had reached levels higher than observed at any other point in 2018–2022, although data from this week are preliminary (Figure 1). Percentages of ED visits with asthma/RAD among children and adolescents aged 5–17 years during these weeks were also qualitatively higher than those during 2020–2021 but were similar to what was reported during 2018–2019 (Supplementary Figure 1, https://stacks.cdc.gov/view/cdc/121524). These observations were consistent when assessing numbers of ED visits with ARI and asthma/RAD rather than percentages (Supplementary Figure 2, https://stacks.cdc.gov/view/cdc/121525). By week 37, the percentage of ED visits with asthma/RAD for either age group had exceeded, at some recent point, levels observed at any time during 2018–2021 in most Health and Human Services regions (Supplementary Figure 3, https://stacks.cdc.gov/view/cdc/121526).

**FIGURE 1:**
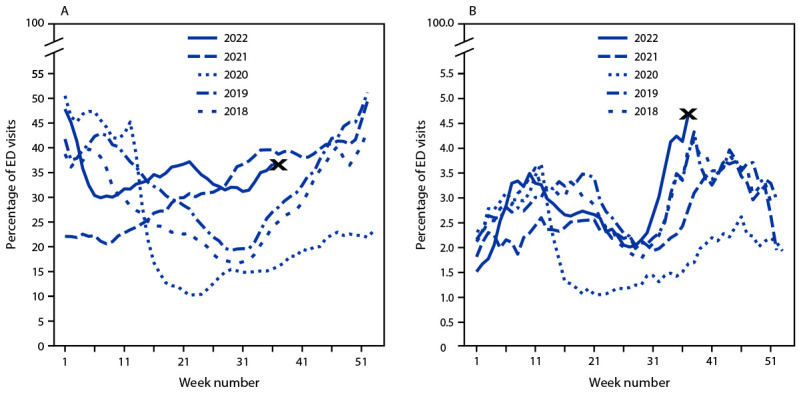
Weekly trends in the reported percentage of emergency department visits associated with acute respiratory illness (A) and asthma/reactive airway disease (B), in children aged 0–4 years, by age group and year — National Syndromic Surveillance Program, United States, January 2018–September 2022* **Abbreviation:** ED = emergency department. * The last reporting week (week 37) ended on September 17, 2022; data from this week are considered preliminary.

The percentage of positive RV/EV nucleic acid amplification test results in NREVSS has been elevated during late summer and early fall during 2014–2022 except in 2020 (Figure 2), and particularly high rates were noted either in late spring or late summer during years with increased EV-D68 detections in the United States (2014, 2016, and 2018). The weekly percentage of positive RV/EV test results in 2022 appears to be increasing at a rate comparable to that in past EV-D68 outbreak years: the percentage of positive RV/EV test results approximately doubled from week 32 (15.8%) to 35 (31.4%), which was the fourth highest value observed for that week after 2014 (41.5%), 2018 (34.4%), and 2015 (31.7%).

**FIGURE 2:**
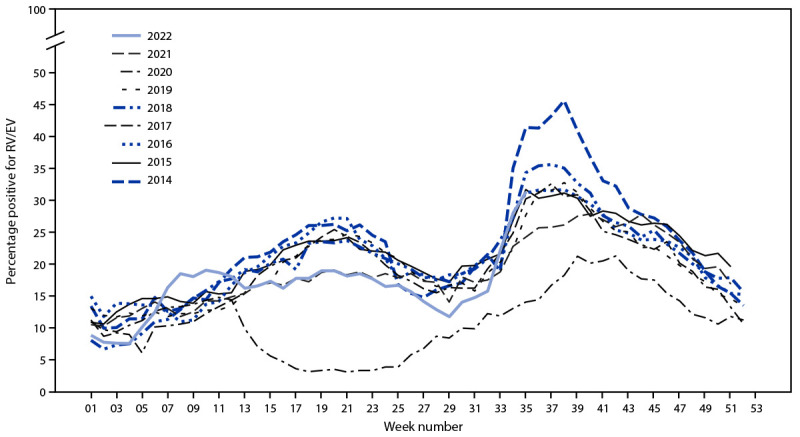
Weekly trends in the reported percentage of positive rhinovirus/enterovirus nucleic acid amplification test results, by year — National Respiratory and Enteric Virus Surveillance System, United States, January 2014–August 2022*^,†^ **Abbreviation:** RV-EV rhinovirus/enterovirus. * The last complete reporting week (week 35) ended on September 3, 2022. ^†^ Enterovirus D68 detections were high during 2014, 2016, and 2018.

During March 1–September 20, 2022, NVSN enrolled 5,633 children and adolescents with ARI seeking emergency care or requiring hospitalization. Testing is ongoing; however, as of September 20, 2022, RV/EV was detected in 1,492 (26.4%) of these patients, among whom 260 (17.4%) had a positive EV-D68 test result. The percentage of positive EV-D68 test results among children and adolescents with ARI and positive RV/EV test results increased to 56% during week 32 (Figure 3). The percentage of positive EV-D68 test results during July and August 2022 was higher than that during the same months of 2017 and 2019–2021 and similar to peak levels observed in 2018. The number of EV-D68 detections and rates of increase varied by geographic location of sentinel sites (Supplementary Figure 4, https://stacks.cdc.gov/view/cdc/121527). The median age of the 260 pediatric patients in NVSN with EV-D68 detected was 2.6 years (IQR = 0–15 years), and the most common signs and symptoms were shortness of breath or rapid shallow breathing, wheezing, cough, and nasal congestion.

**FIGURE 3:**
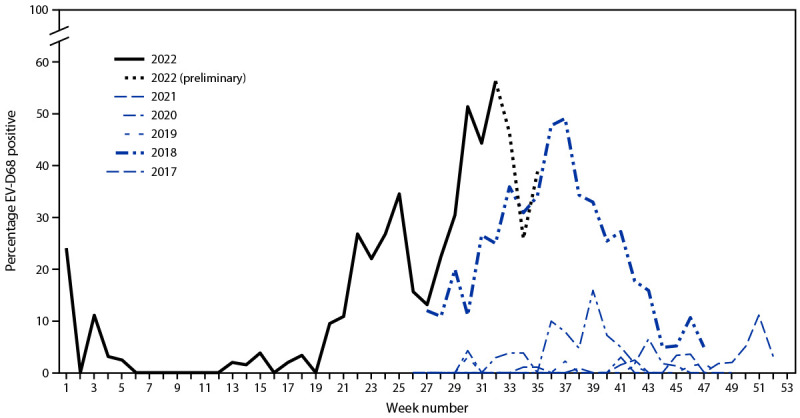
Weekly trends in reported percentage of positive enterovirus D68 test results among children and adolescents aged <18 years with acute respiratory illness and positive rhinovirus/enterovirus test results who received care in the emergency department or inpatient units — New Vaccine Surveillance Network,* United States, 2017–2022^†^ **Abbreviation:** EV-D68 = enterovirus D68. * The seven sites in the New Vaccine Surveillance Network are located in Kansas City, Missouri; Rochester, New York; Cincinnati, Ohio; Pittsburgh, Pennsylvania; Nashville, Tennessee; Houston, Texas; and Seattle, Washington. Two sites do parallel testing with a pan-rhinovirus and EV-D68 assay; fives sites do sequential testing with a pan-rhinovirus and pan-enterovirus assay or a rhinovirus/enterovirus assay, followed by an EV-D68 assay. All sites use the same CDC-developed EV-D68 reverse-transcription–polymerase chain reaction assay. ^†^ Testing for EV-D68 occurred at all seven sites during July–October 2017 and during July–November 2018–2020. Year-round testing began at most sites in July 2021 and was fully implemented at all sites during June 2022. EV-D68 testing windows in NVSN have changed over time, limiting annual comparisons outside of these windows. Retrospective testing is still in process for 2021 and early 2022, and data are current as of September 22, 2022. Weeks 33–35 are subject to delays in reporting.

## Discussion

Using data from three separate surveillance systems, this analysis found an increase in medically attended ARI and asthma/RAD exacerbations in children and adolescents during summer 2022. This rise might be attributable, in part, to increased RV/EV circulation and specifically circulation of EV-D68. In 2014, a widespread EV-D68 outbreak in the United States caused similar increases in medically attended severe respiratory illnesses and asthma exacerbations and was associated with an increase in AFM cases (*2*,*3*). Surveillance efforts for EV-D68 were enhanced after this outbreak, including the establishment of active, prospective sentinel surveillance (*2*,*5*,*6*). The seasonality of EV-D68 and associated AFM cases remains poorly characterized, but biennial peaks occurred in 2014, 2016, and 2018, before the COVID-19 pandemic (*7*). Ongoing surveillance is necessary to understand when and where future circulation and EV-D68–associated severe illness might occur, given the potential changes in virus circulation and population immunity related to COVID-19 mitigation measures (*1*).

The findings in this report are subject to at least five limitations. First, differences in surveillance catchment populations and representativeness limit direct comparisons across systems and generalizability of findings. Second, delays in reporting vary by system and might result in underestimates of recently reported data. Third, in the ED data, ARI is a broad definition designed to capture all diagnoses related to respiratory illness, including SARS-CoV-2, influenza, pneumonia, and cough, potentially limiting specificity for identifying visits with EV-D68-associated respiratory illnesses. Fourth, the COVID-19 pandemic likely affected health care–seeking behaviors and testing practices in multiple ways; these differences could affect comparability of recent data to 2019 and previous years. Finally, comparable NSSP data on hospitalizations or trends before 2018 are unavailable, as are NVSN data before 2017.

Clinicians are advised to consider EV-D68 as a possible cause of severe respiratory illness in children and adolescents, particularly those with wheezing or who require respiratory support. Health care facilities should be prepared for possible increases in pediatric health care use associated with severe EV-D68–associated respiratory illness (*8*). Past increases in EV-D68 circulation were also associated with increased reports of AFM.^§§§^ Providers should have a high index of clinical suspicion for AFM in patients with acute flaccid limb weakness, neurologic signs and symptoms, or neck or back pain who have a recent history of respiratory illness or fever. Children with AFM can experience rapid progression of weakness and should be promptly hospitalized and referred to specialty care.^¶¶¶^ Given the detection of a paralytic polio case and wastewater samples positive for poliovirus in New York during summer 2022 (*9*), clinicians should also test for poliovirus infection in patients suspected of having AFM because of the clinical similarity to acute flaccid paralysis caused by poliovirus. Providers should immediately report possible AFM cases and acute flaccid paralysis cases suspected of polio to local and state health departments and coordinate with health departments and CDC for testing protocols.****

**Summary**

**What is already known about this topic?**

Enterovirus D68 (EV-D68) caused biennial outbreaks of severe respiratory illness and acute flaccid myelitis (AFM) in the United States in 2014, 2016, and 2018.

**What is added by this report?**

After an extended period of low EV-D68 circulation during the COVID-19 pandemic, surveillance data suggest increased detection of rhinovirus/enterovirus and EV-D68, concurrent with increased emergency department visits by children and adolescents with acute respiratory illness and asthma/reactive airway disease during summer 2022.

**What are the implications for public health practice?**

Clinicians should consider EV-D68 as a possible cause of acute respiratory illness and AFM in children and adolescents this fall and be aware of guidance for prompt testing and referral for patients with suspected AFM.

## Footnotes

*
https://emergency.cdc.gov/han/2022/han00474.asp

† Additional challenges are that 1) RV/EV testing is available primarily as part of respiratory viral panels, which are expensive, limiting widespread clinical use; 2) RV/EVs include multiple virus types that cannot be distinguished clinically or in most respiratory viral panels; and 3) clinical facilities with EV-D68–specific testing are uncommon, and test use is primarily limited to RV/EV–positive specimens.

§ NSSP is a network comprising CDC representatives, state and local health departments, and academic and private sector health partners jointly collecting and sharing electronic patient encounter data. NSSP’s BioSense Platform includes approximately 6,000 health care facilities with coverage for 49 states and the District of Columbia. NSSP includes ED visit data from approximately 71% of U.S. EDs.

¶ The CDC “Broad Acute Respiratory Discharge Diagnosis (DD) v1” definition identifies ED visits associated with general respiratory infections (e.g., influenza, respiratory syncytial virus, or coronavirus) as well as general respiratory illness such as cough or pneumonia. These are identified in *International Classification of Diseases, Tenth Revision, Clinical Modification* (ICD-10-CM) and *International Classification of Diseases, Ninth Revision, Clinical Modification* (ICD-9-CM) discharge diagnoses.

** The syndrome definition used for asthma/RAD is the ESSENCE “CDC Asthma Chief Complaint/Discharge Diagnosis (CCDD) v1” and contains query criteria for terms related to asthma, bronchospasm, and reactive airway disease and selected misspellings appearing in the chief complaint. Discharge diagnosis codes were also included in this query for ICD-10-CM and SNOMED CT.

†† For extended historic timeseries and analysis, NSSP applies data quality filters to account for fluctuations attributable to site and facility onboarding and outages. Data quality filters for these time series include an indicator of an Emergency Patient Class, an average weekly percentage of visits with informative discharge diagnosis ≥70%, and a data quality coefficient of variance ≤30 over the last 4 years to date.

§§ Untyped RV/EV results are reported to NREVSS, a voluntary, passive surveillance system of 473 U.S. public health, clinical, and reference laboratories, which report weekly aggregate RV/EV nucleic acid amplification tests performed and the number of positive RV/EV detections. RV/EV results were first reported to NREVSS in July 2006. NREVSS only collects untyped RV/EV results.

¶¶ NVSN includes seven U.S. medical centers that perform active surveillance for pediatric ARI. EV-D68 testing occurred July–October 2017, July–November of 2018–2020, and July 2021 onwards, when year-round testing began. Retrospective testing is still underway for 2021 and early 2022. Two sites conduct parallel testing with a pan-RV and EV-D68 assay; five sites do sequential testing with either a pan-RV, pan-EV, or RV/EV test followed by an EV-D68 assay. All sites use the CDC-developed EV-D68 reverse transcription–polymerase chain reaction assay.

*** 45 C.F.R. part 46, 21 C.F.R. part 56; 42 U.S.C. Sect. 241(d); 5 U.S.C. Sect. 552a; 44 U.S.C. Sect. 3501 et seq.

†††
https://emergency.cdc.gov/han/2021/han00443.asp

§§§ As of September 21, 2022, among 45 patients under investigation, 15 (33%) cases of AFM had been confirmed in ten states during 2022. In years of increased EV-68 circulation, 120 (2014), 153 (2016), and 238 (2018) cases of AFM had been reported in each year, compared with 22 (2015), 38 (2017), 47 (2019), 33 (2020), and 28 (2021) in years of low EV-68 circulation. https://www.cdc.gov/acute-flaccid-myelitis/cases-in-us.html

¶¶¶
https://www.cdc.gov/acute-flaccid-myelitis/hcp/clinicians-health-departments/clinical-presentation.html

****
https://www.cdc.gov/acute-flaccid-myelitis/hcp/specimen-collection.html
